# Relationship between Physical Disability and Depression by Gender: A Panel Regression Model

**DOI:** 10.1371/journal.pone.0166238

**Published:** 2016-11-30

**Authors:** Jin-Won Noh, Young Dae Kwon, Jumin Park, In-Hwan Oh, Jinseok Kim

**Affiliations:** 1 Department of Healthcare Management, Eulji University, Seongnam, Korea; 2 Global Health Unit, Department of Health Sciences, University Medical Center Groningen, University of Groningen, Groningen, The Netherlands; 3 Department of Humanities and Social Medicine, College of Medicine and Catholic Institute for Healthcare Management, the Catholic University of Korea, Seoul, Korea; 4 National Institutes of Health Clinical Center, Bethesda, MD, United States of America; 5 Department of Preventive Medicine, Kyung Hee University School of Medicine, Seoul, Korea; 6 Department of Social Welfare, Seoul Women’s University, Seoul, Korea; Hangzhou Normal University, CHINA

## Abstract

**Background:**

Depression in persons with physical disabilities may be more common than in the general population. The purpose of this study was to examine the relationship between physical disability and depression by gender among adults, using a large, nationally representative sample.

**Methods:**

This study used data from the Korean Longitudinal Study of Aging, Wave one through four, and ran a series of random effect panel regression models to test the relationship between physical disability status and depression by gender. We tested the moderating effect of gender on the relationship between disability status and depression level by examining the significance of the cross-product term between disability status and gender.

**Results:**

After controlling for self-rated health, marital status, employment status, education, and age, subjects who were female or diagnosed as having any disability presented higher levels of depression scores. Further, the difference in terms of their depression level measured by Center for Epidemiologic Studies Short Depression Scale (CES-D 10) scores between those who were diagnosed as having any disability and those who were not was greater for females than for their male counterparts.

**Conclusion:**

This study reaffirmed that disability is the risk factor of depression, using longitudinal data. In addition, female gender is the effect modifier rather than the risk factor. The effect of gender in the non-disability group, mostly composed of older persons, is limited. On the contrary, the female disability group showed more depressive symptoms than the male disability group. The gender difference in the disability group and the role of culture on these differences need further research.

## Introduction

Depression is an increasingly prevalent public health concern, affecting an estimated 350 million people globally [1]. The 2011 World Mental Health Survey of 17 countries found that approximately one in 20 people has experienced a depressive episode [2]. Depressive symptoms have a considerable impact on mortality risk for suicide and cardiovascular and other diseases as well as impaired cognitive and social functioning [3]. In this respect, the burden of disease and the associated economic costs stemming from depression are great [1, 3].

Physical disability is found to be related to depressive symptoms. People with physical disability experience multiple risk factors for depressive symptoms, including stereotypic social and personal attitude; abuse; loss of roles; and stressors related to poverty, environmental barriers, and/ or lack of access to appropriate health care [4,5]. Substantial evidence shows that people living with physical disabilities are at least three times more likely to experience depression compared to the general population [4,6–8].

Gender was hypothesized as a potential moderator of depression [9]. A growing body of literature has examined the association between gender and depression, with controversial results. Much research supports the possibility that women are more likely to be depressed than men [10–15]; however, some studies [16–19] reported that there is no clear relationship between gender and depression among older adults.

Given the probable association between depression and gender, the discrepancy could be attributable to the differences in confounding the socio-demographic and health-related factors. Research reports several factors associated with the higher risk of depression, including a lack of cohabitants [14,17,20], low education attainment [15,17,20], low income [14,17], low health status [10], and poor physical function [14].

Regarding the relationship between depression and physical disabilities by gender, several studies reported that women living with disabilities may be more likely to experience more depressive symptoms compared to their male counterparts. Depression is estimated to affect more women with disabilities, with estimates ranging from 30% [21] to 59% [4,6,8]. According to Healthy People 2010 [8], women with disabilities were more likely to show feelings of sadness, unhappiness, or depression, which discouraged them from being active, compared to women without disabilities.

As for exploring the relationships between depression and disability in adults, few studies have explored the influence of gender on the association between the two. Therefore, there is little empirically based evidence suggesting that clinical practice is different in the psychosocial rehabilitation and community reintegration of women and men with disabilities [5]. Most of the existing studies have primarily focused on Western countries, and in-depth investigations among Asian populations are limited in adults [22]. In addition, the majority of studies exploring the relationships among depression, physical disability, and gender were limited to a single point in time. In this study, panel data analysis would provide more accurate inference of model parameters, capturing the complexity of human behavior, than single cross-sectional data. It also would simplify computation and statistical inference, allowing for more information on the causal direction of the association [23]. The purpose of this study was to investigate the relationship between physical disability and depression by gender, using nationally representative data and a panel regression model.

## Methods

### Data and Subjects

We used the data from the Korean Longitudinal Study of Ageing (KLoSA), which was collected by the Korean Labor Institute and funded by the Korean Ministry of Labor. This survey was performed using computer-assisted personal interviewing of individuals aged 45 years or older to obtain comprehensive data, including socio-demographic characteristics, employment status, health status, finance, and family and social networks. KLoSA collected data every other year beginning in 2006, and data from 2006 (*N* = 10,254), 2008 (*N* = 8,688), 2010 (*N* = 7,920), and 2012 (*N* = 7,486) were used in the present study. This study was approved by the Institutional Review Board of the corresponding author’s institution, Seoul Women’s University (SWU IRB 2015A-33), and informed consent was waived because the data was obtained from a public database (http://survey.keis.or.kr).

### Variables

The Korean version of CES-D 10 was used to assess the level of depressive symptoms. Answers to the questions in the Korean version of CES-D 10 were composed of a four-point frequency scale [0 = occasionally (less than one day); 1 = sometimes (from one to two days); 2 = often (from three to four days); 3 = at all times (from five to seven days)]. The total score of the 10 items, ranging from 0 to 30, was used as a depression score in this analysis, with higher score indicating more severe depressive symptoms. Cronbach’s alpha in this sample ranged from .822 to .861 throughout the study period.

The KLoSA study participants were asked whether they had any physical disability diagnosed by doctors; their answers were used to measure disability status in this study. Types of physical disability asked in the KLoSA were physical disability, brain lesions, visual handicap, hearing impairment, language disorder, kidney lesions, and cardiac lesions. Other covariates such as marital status [1 = married; 0 = not married (i.e., divorced, parted by death, separated, or single)], level of education (college or higher, high school graduate, middle school graduate, and elementary school or lower), employment status (1 = employed; 0 = unemployed) were included in the analysis. Participants’ self-rated health status was measured using a five-point Likert scale (1 corresponding to “very good” and 5 to “very bad”) and included in the analysis as well.

### Statistical Analysis

Descriptive analyses of the data were conducted to examine the sample characteristics. Because the panel data were repeatedly measured, we estimated a set of random effect panel regression models [24] to test the relationship between the level of depression and disability status among Korean adults. Analysis models in this study could be expressed in the following equation where u_j0_ referred to random intercept, γ_1_ to effect size of moderation effect between gender and disability on depression status, and X’s to other covariates in the model:

$$Y_{ij}=\gamma_{00}+\sum_{k}\gamma_{k0}X_{ij}^{k}+\gamma_{1}{\left(gender\right)}_{ij}\times {\left(disability\right)}_{ij}+u_{j0}+e_{ij}$$

We tested the moderating effect of gender on the relationship between the level of depression and disability status by examining the significance of the cross-product term between disability status and gender in the same model. We used Stata version 13.1 (StataCorp LP, College Station, TX, USA) to estimate the analysis models.

## Results

Socio-demographic and other relevant characteristics of the study sample are summarized in Table 1. In year 2006, there were 10,254 adults aged between 45 and 105 years [mean (SD) = 61.7 (11.1)] of which 5,792 (56.5%) were female. Over three quarters (77.8%) of the study participants were married and about 38% reported that they were employed. About half the participants (47%) reported elementary school graduate or lower; 16% were middle school graduates; 26% were high school graduates; and 10% had a college or graduate degree. Over 30% of the participants reported their health status as very bad (7%) or bad (24%), whereas 34% reported good and 4% very good health. About 6% of the participants reported diagnosed physical disability (Table 1).

**Table 1:** Characteristics of Study Sample Stratified by Gender.

Variable	Total		Male		Female		
	(*N* = 10,254)		(*N* = 4,462)		(*N* = 5,792)		*t*
	Mean	(SD)	Mean	(SD)	Mean	(SD)	
Age (years at 2006)	61.7	(11.1)	61.2	(10.6)	62.1	(11.5)	4.04^*^
CES-D 10 scores							
Year 2006 (*N* = 10,180)	6.65	(5.13)	5.99	(4.75)	7.16	(5.35)	11.5^*^
Year 2008 (*N* = 8,636)	7.45	(5.66)	6.58	(5.25)	8.13	(5.86)	12.8^*^
Year 2010 (*N* = 7,868)	7.53	(5.64)	6.89	(5.35)	8.02	(5.80)	8.8^*^
Year 2012 (*N* = 7,423)	7.40	(5.46)	6.82	(5.18)	7.84	(5.62)	8.0^*^
	*N*	%	*N*	%	*N*	%	χ^2^
Marital status							883.4^*^
Married	7,971	77.8	4,089	91.7	3,882	67.0	
Not married	2,281	22.3	372	8.3	1,909	33.0	
Education							940.7^*^
Elementary school or lower	4,824	47.1	1,420	31.9	3,404	58.8	
Middle school	1,657	16.2	759	17.0	898	15.5	
High school	2,708	26.4	1,501	33.7	1,207	20.9	
College or higher	1,.57	10.3	779	17.5	278	4.8	
Employed							1.0
Not employed	6,366	62.1	2,795	62.6	3,571	61.7	
Employed	3,888	37.9	1,667	37.4	2,221	38.4	
Self-rated health status							315.7^*^
Very bad	700	6.8	251	5.6	449	7.8	
Bad	2,487	24.3	793	17.8	1,694	29.3	
Neutral	3,208	31.3	1,358	30.4	1,850	31.9	
Good	3,502	34.2	1,845	41.4	1,657	28.6	
Very good	357	3.5	215	4.8	142	2.5	
Disability status							80.4^*^
Diagnosed as disable	649	6.3	392	8.8	257	4.4	
No diagnosis	9,605	93.7	4,070	91.2	5,535	95.6	

*p < .05

SD, standard deviation; CES-D 10, Center for Epidemiologic Studies Short Depression Scale

**Fig 1:**
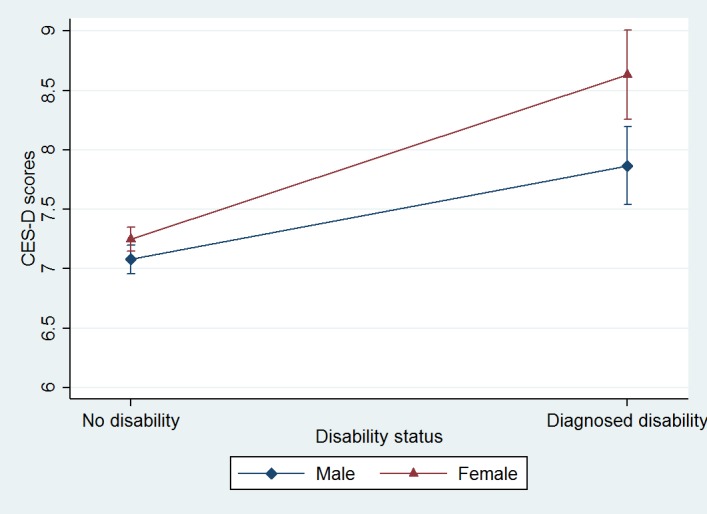
Relationship Between Depression And Disability Status by Gender. CES-D, Center for Epidemiologic Studies Depression Scale.

Table 2 summarizes the random effect panel model of depression measured by CES-D 10 with disability status and gender as explanatory variables after controlling for socio-demographic covariates. Results from Model 1 showed that, after controlling for other covariates such as self-rated health status, marital status, employment status, education, and age, those who were female [B (SE) = 0.21 (0.08), *p* = .008] or diagnosed with physical disability by doctors [B (SE) = 1.05 (0.13), *p* < .001] presented higher scores in level of depression (Table 2). Fig 1 illustrates that the difference in terms of their CES-D 10 scores between those who were diagnosed as having any physical disability and those who were not was greater for females than for males (Fig 1). A further test of the interaction effect between disability status and gender in terms of the association with respondents’ depression scores in Model 2 of Table 2 revealed that the association between physical disability status and depression score was stronger for females than for their male counterparts [B (SE) = 0.60 (0.36), *p* = .022].

**Table 2:** Random Effect Panel Regression Models of CES-D 10.

	B	SE (B)	95% CI	B	SE (B)	95% CI
Self-rated health status	–1.84^***^	0.03	(–1.90, –1.78)	–1.84^***^	0.03	(–1.9, –1.78)
Married	–1.34^***^	0.09	(–1.52, –1.17)	–1.34^***^	0.09	(–1.52, –1.17)
Employed	–0.15^**^	0.05	(–0.25, –0.05)	–0.15^**^	0.05	(–0.25, –0.05)
Education	–0.19^***^	0.04	(–0.27, –0.11)	–0.19^***^	0.04	(–0.27, –0.11)
Age	0.05^***^	0.00	(0.05, 0.06)	0.05^***^	0.00	(0.04, 0.06)
Disability	1.05^***^	0.13	(0.80, 1.31)	0.79^***^	0.17	(0.44, 1.13)
Female	0.21^**^	0.08	(0.06, 0.37)	0.17^*^	0.08	(0.00, 0.33)
Disability X Female				0.60^*^	0.26	(0.09, 1.11)
Intercept	10.68^***^	0.35	(9.99, 11.36)	10.72^***^	0.35	(10.03, 11.41)
	Variance	Chi^2^		Variance	Chi^2^	
Random intercept	8.68	4742.78^***^		8.67	4732.20^***^	

* *p* < .05

** *p* < .01

*** *p* < .001

CES-D 10, Center for Epidemiologic Studies Short Depression Scale; SE, standard error; CI, confidence interval

## Discussion

The association among gender, disability, and depression has attracted attention [11]. Disability and depression have been known to be reciprocal risk factors [25]. Furthermore, the association between gender and depression has shown controversial results, and some researchers asserted that gender and depression varied with life span [22]. This study, using a nationally representative sample, shows that being female and disabled is associated with depression as measured by CES-D 10 scores. Bad self-rated health, unmarried status, unemployment, low education status, and increasing age are also associated with higher CES-D 10 scores. When the interaction between disability and gender is considered, it is statistically significant, and being female shows a further borderline significance. The effect of other factors such as self-rated health, unmarried stats, and low education appears be similar to a non-interaction model.

Disability is known to be a risk factor for depression [25–27]. The association between disability and depression could be found in various studies, from those using symptom scores to those using major depression cases [25], and from cross-sectional studies to longitudinal studies. One possible explanation of this association is that disability functions as the stressful condition. In this view, new-onset disability requires patients to adjust and could exert chronic strain when it prevents their daily activities. These factors increase the risk of depression. The disability could also be the symptoms of depression. This hypothesis is pertinent in cases of functional disability. In this study, disability is defined by the self-reported doctor-diagnosed disability, verified by legal registration; the meaning of disability in our study is thus more official than functional [28]. Because we did not differentiate between new-onset disability and an existing one, it is hard to identify the disability as chronic strain or a new stressful condition. However, our study also supports disability as a risk factor for depression when measured by symptom scores in a longitudinal study.

Another important finding observed in this study is the modifying effect of gender. The relationship between gender and disability is the debate [22]. For example, the relationship between gender and depression could vary with life span [22]. It is known that there is no difference in the prevalence of depression between male and female during childhood. Boys showed even higher prevalence of depression symptoms than girls, but the prevalence of depression is higher in females than in males in an adolescent group. The higher prevalence of depression in female adults displayed a consistency with the findings in the adolescent group. From the age of 55 or 65, though, the gender difference of depression varies by the study [22]. Some studies found little or no gender difference [17,22]; other studies found clear gender differences [10,11,15]. This lack of clarity could be associated with study design such as sample size and measures of depression. Cultural characteristics could be related to this lack of clarity, too [22]. This study showed that although the female group’s CES-D 10 score is higher than the male group’s, the effect of gender appears in the disability group only when the interaction effect is considered. These findings support the previous Korean study showing that gender effect is not clear [17]; it showed that the prevalence of depression in females is higher than that of males in the 65-year and older group, but the effect of gender is not clear in the multivariable model. They argued that the risk factors that could be dominant in females, such as cognitive impairment, older age, or low socioeconomic status, endorsed the gender difference of depression rather than gender itself. Our results also showed that in the non-disability group, the effect of gender has only a borderline significance; gender could be considered a modifier of disability. In the disability group, the CES-D 10 scores of the female group were much higher than those of the male counterparts even when other risk factors were adjusted. Perceived social support, sense of control and self-esteem is postulated as to mediate the effect of functional disability on depressive symptoms though the study on gender difference among disabled is very scarce [29]. The amplified depressive symptoms of female disability group may be related with reduced access to material and social conditions of female compared with male counterpart [30]. The increasing prevalence of depression is observed in the elderly also, but it is not clear that the result is from other risk factors such as low socioeconomic status or disability or aging itself. A study that was conducted in Germany reported that the depression prevalence was lowest in the 65 to 69 age group and highest in the 53 to 59 group, which faced retirement [15]. Previous Korean studies, which included only 65-year and older populations, found that the odds of depression were highest in the 75 to 79 age group in multivariable analysis [17]. The odds of depression in the 85-year and older age group does not differ from that of the 65 to 69 age group. On the contrary, our results showed that the effect of aging was closely related to higher CES-D 10 scores even in multivariable analysis. The study design that measured depression, or the covariates that were considered, and the study period and the age group that it included, could be correlated with the difference of results.

The previous Korean study also reported that low income, low education, lack of cohabitant, and illiteracy were risk factors for depression [17]. The result of our study showed similar results. In the case of unmarried status, the previous study did not show a statistical significance. Our study did not consider cohabitant as a variable due to data limitation, but marital status could be considered a proxy for cohabitant. Then it might suggest that cohabitant rather than spouse could be the preventive factor against depression, according to the previous Korean study, but many studies report that bereavement is closely related to depression, so further research needs to be conducted [10]. Low education and illiteracy appear to result in poor social support [17]. The German study also showed the association of low education and comorbidity with depression in line with our results [15]. Bad self-rated health was also known to be a risk factor for depression, which supports our study [10].

Our study has several limitations. First, we used the disability variable defined as a dichotomous one. This could cause an issue because previous studies indicated that types or severity of disability were associated with the level of depression [31,32]. Even though the KLoSA collected respondents’ status regarding physical disability, brain lesions, visual handicap, hearing impairment, language disorder, kidney lesions, and cardiac lesions, the prevalence of all the types but physical disability were too small, or smaller than 0.1%, to provide reliable estimation of each type’s effect on the level of depression. On the other hand, we also checked a potential effect of severity of disability by conducting a sensitivity analysis (results not shown), which did not indicate a potential severity effect on the relationship between disability and depression.

Second, we could not consider all possible risk factors for depression due to the restraint of data we used. For example, prior depression or sleep disturbance, smoking, and head trauma are known to be risk factors for depression [10,17]. Physical activity and moderate alcohol use, however, are related to low risk of depression [17]. If these factors could have been considered, we could grasp the relationship of gender, disability, and depression better, especially in older persons. Furthermore, although we measure depression by CES-D 10 score, if other indexes, such as a short-form scale of geriatric depression were used, the relationship could be varied [17]. Although the index is different in other studies, our result is in line with other studies. Also, because we defined disability as doctor-diagnosed, the effect of disability on depression could be underestimated.

Despite these limitations, this study showed that disability is closely related to depression, using nationally representative samples in Korea. This result confirms the role of disability as the risk factors of depression, using longitudinal data. We found, too, that female gender performed as an effect modifier in the disability group rather than as a risk factor. The female disability group showed higher CES-D 10 scores than the male disability group. The effect of gender in non-disability groups which were mostly composed of aged people, is restricted. Though the study on the gender difference is numerous, the study on gender difference of disabled is very scarce and we found that the relationship between disability and depression is amplified among female. Therefore, when intervention that could prevent depression in the disabled will be implemented, the gender difference in disability groups should be considered.
